# Investigation of the Chemical Changes from Crude and Processed Paeoniae Radix Alba-Atractylodis Macrocephalae Rhizoma Herbal Pair Extracts by Using Q Exactive High-Performance Benchtop Quadrupole-Orbitrap LC-MS/MS

**DOI:** 10.1155/2014/170959

**Published:** 2014-05-04

**Authors:** Gang Cao, Qinglin Li, Hao Cai, Sicong Tu, Baochang Cai

**Affiliations:** ^1^College of Pharmacy, Nanjing University of Chinese Medicine, Nanjing 210023, China; ^2^Research Center of TCM Processing Technology, Zhejiang Chinese Medical University, Hangzhou 310053, China; ^3^Zhejiang Cancer Hospital, Hangzhou 310022, China; ^4^Faculty of Medicine, University of New South Wales, Sydney, NSW 2031, Australia

## Abstract

The Paeoniae Radix Alba-Atractylodis Macrocephalae Rhizoma herbal pair is mainly used for regulating the functions of liver and spleen, benefiting *qi*, and nourishing blood. However, the bioactive compounds for the pharmacological activities of the crude and processed Paeoniae Radix Alba-Atractylodis Macrocephalae Rhizoma herbal pair extracts are still unclear to date. In the present study, Q Exactive high-performance benchtop quadrupole-Orbitrap LC-MS/MS was applied to identify the complicated components from crude and processed Paeoniae Radix Alba, crude and processed Atractylodis Macrocephalae Rhizoma, and their crude and processed herbal pair extracts. 123 and 101 compounds were identified in crude and processed Paeoniae Radix Alba samples, respectively. Meanwhile, 32 and 26 compounds were identified in crude and processed Atractylodis Macrocephalae Rhizoma samples, respectively. In the crude and processed Paeoniae Radix Alba-Atractylodis Macrocephalae Rhizoma herbal pair extracts, co-decoction could significantly change the chemical composition of Paeoniae Radix Alba and Atractylodis Macrocephalae Rhizoma in solution. The developed method may provide a scientific foundation for deeply elucidating the processing and compatibility mechanism of Paeoniae Radix Alba and Atractylodis Macrocephalae Rhizoma.

## 1. Introduction

Traditional Chinese medicine (TCM) processing is regarded as a pharmaceutical technology based on TCM theory, the requirements of different syndrome treatment, the quality nature of medicine, and different demands of clinical dispensing and preparations [1]. It is one of the characteristics in application of TCM. The compatible components of prescription are composed of prepared Chinese crude drugs after TCM processing.

The prescription compatibility and TCM processing are not only two major features of clinical medication in TCM, but are also critical to distinguish TCM from natural medicine. The research on structural features, compatible effect, and material basis of the herbal pair is the important support in the study of the prescription compatibility since the herbal pair is the minimum unit in prescription of TCM [2, 3]. They play a guidance and significant role in reveal of the compatibility rule and the scientific connotation. The herbal pair compatibility theory can explain the relationship of the prescription compatibility to some extent. The research on the relationship between the herbal pair compatibility and the prescription compatibility contributes to the elucidation of the prescription compatibility mechanism and the action mechanism of treatment. There are many herbal pairs commonly used in the clinical practice of TCM, such as the herbal pairs of Paeonia Lactiflora-Liquorice, Ginseng-Aconite, and Aconite-Rhizome Zingiberis [4, 5] besides the Paeoniae Radix Alba-Atractylodis Macrocephalae Rhizoma herbal pair frequently used in all China dynasties [6, 7]. Paeoniae Radix Alba nourishes blood and liver, and Atractylodis Macrocephalae Rhizoma helps invigorate spleen and eliminate dampness [8–12]. Thus, the compatibility of these two medicines could help achieve the goal of purging wood from the earth, regulating the functions of liver and spleen, benefiting* qi,* and nourishing blood [13–15]. Although the compositions of these two medicines have been extensively studied, the appropriate processing method of them, such as frying, which is believed by the practitioners of traditional medicine to have the effects for enhancing the efficacy of the medicine, and their underlying compatibility mechanism are still under investigation.

The objective of this study is to investigate the qualitative, preprocessing, and postprocessing changes in the composition and compatibility of Paeoniae Radix Alba and Atractylodis Macrocephalae Rhizoma by using Q Exactive hybrid quadrupole-Orbitrap mass spectrometer combined with high-performance quadrupole precursor selection with high-resolution and accurate-mass Orbitrap detection. The work could serve as a theoretical basis for the development of medicines from Paeoniae Radix Alba and Atractylodis Macrocephalae Rhizoma, and the reasonable clinical medication. Furthermore, it provides new insights into the investigation of the herbal pair and for the study of the appropriate processing method for Chinese herbal medicines and their underlying compatibility mechanism.

## 2. Experimental

### 2.1. Chemicals, Solvents, and Herbal Materials

Paeoniae Radix Alba and Atractylodis Macrocephalae Rhizoma samples were acquired from Zhejiang suppliers. All of these herbal samples were authenticated by Professor Jianwei Chen (College of Pharmacy, Nanjing University of Chinese Medicine). HPLC-grade acetonitrile and formic acid were obtained from Merck (Darmstadt, Germany). Deionized water was purified using the Milli-Q system (Millipore, Bedford, MA, USA). All other reagents and chemicals were analytical grade.

### 2.2. Preparation of the Sample Solutions

The dried and powdered samples of crude and processed Paeoniae Radix Alba, crude and processed Atractylodis Macrocephalae Rhizoma, and their crude and processed herbal pair extracts (1 : 1, g/g) were prepared. A total of 2.0 g of each sample powder was accurately weighed and transferred into a 50 mL round bottom flask with 20 mL of 70% methanol aqueous solution (v/v) and refluxed in a 80°C water bath for 1 h. The filtrate was collected after filtration and the residue was then refluxed with 20 mL of 70% methanol aqueous solution in a 80°C water bath for 1 h, the filtrate was collected again after filtration and the residue was removed. Finally, the combined filtrates were treated by rotary evaporation concentration and the resultant residue was dissolved and transferred into a 25 mL volumetric flask with 70% methanol aqueous solution to make it up to a final concentration of 0.08 g·mL^−1^. All solutions were stored at 4°C and filtered through a 0.22 *μ*m filter membrane before injection into the HPLC system.

### 2.3. Liquid Chromatography and Mass Spectrometry

Analyses were performed by using Dionex UltiMate 3000 HPLC system (Dionex, Sunnyvale, CA, USA) with a diode array detector. Detection wavelengths were set at 255 nm. A Thermo Scientific Hypersil Gold C_18_ column (100 mm × 2.1 mm, 1.9 *μ*m) was used with a flow rate of 0.35 mL·min^−1^. The injection volume was 5 *μ*L, and the column temperature was maintained at 30°C. The sample separation was performed according to the previous reports with minor modification [16–18]. The mobile phase was composed of (a) aqueous formic acid (0.1%, v/v) and (b) acetonitrile under following gradient elution: 10–55% B from 0 to 40 min, 55–90% B from 40 to 51 min, 90% B from 51 to 56 min, 90–10% B from 56 to 56.1 min, and 10% B from 56.1 to 60 min. Mass spectrometry was performed on a Q Exactive high-resolution benchtop quadrupole Orbitrap mass spectrometer (Thermo Fisher Scientific, San Jose, USA) using a heated electrospray ionization (HESI-II) source for ionization of the target compounds in positive and negative ion modes. The key parameters were as follows: ionization voltage, +3.0 kV/−2.8 kV; sheath gas pressure, 35 arbitrary units; auxiliary gas, 10 arbitrary units; heat temperature, 300°C; and capillary temperature, 300°C. For the compounds of interest, a scan range of* m*/*z* 150–1500 was chosen. Resolution for higher energy collisional dissociation cell (HCD) spectra was set to 17,500 at* m*/*z* 150 on the Q Exactive.

## 3. Results and Discussion

### 3.1. Identification of the Main Components in Crude and Processed Paeoniae Radix Alba

**Figure 1:**
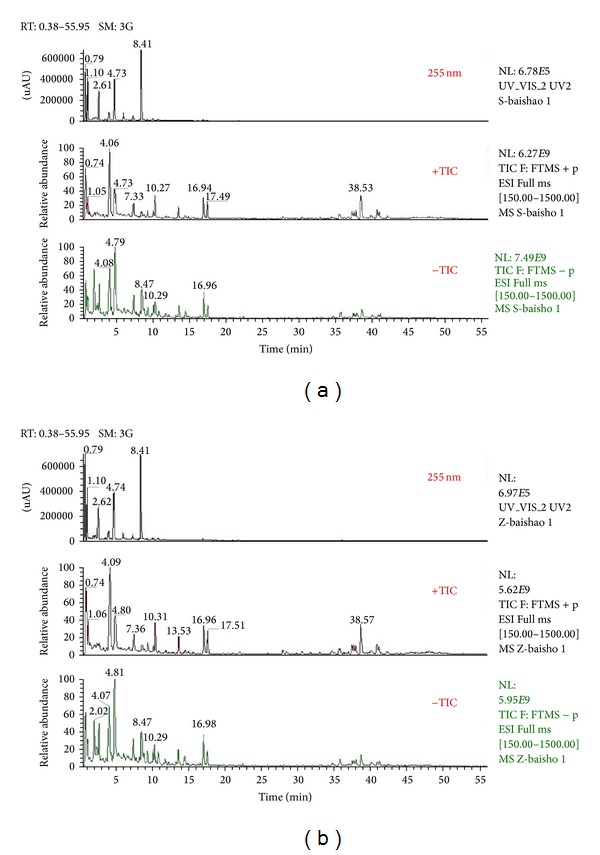
Total ion chromatograms of crude (a) and processed (b) Paeoniae Radix Alba obtained from both positive and negative ion modes.

Tentative identification of the main compounds in crude and processed Paeoniae Radix Alba samples was generated based on elemental composition data determined from accurate mass measurements and comparison with the literature data. The total ion chromatograms of crude and processed Paeoniae Radix Alba samples obtained from both positive and negative ion modes were shown in Figure 1. In the preliminary study, the Q Exactive mass spectrometer was confirmed to be highly selective and sensitive. Under the present chromatographic and MS conditions, 123 and 101 compounds were identified in crude and processed Paeoniae Radix Alba samples, respectively. Compounds 16, 30, 31, 42, 45, 58, 59, 61, 62, 63, 64, 75, 78, 80, 87, 90, 91, 94, 95, 103, 112, and 120 were not detected in processed Paeoniae Radix Alba sample. Meanwhile, the ESI-MS data of crude and processed samples demonstrated that the peak areas of components 8, 113, and 122 varied significantly, and their amounts were dramatically increased in processed sample. The results were shown in Table 1.

**Figure 2:**
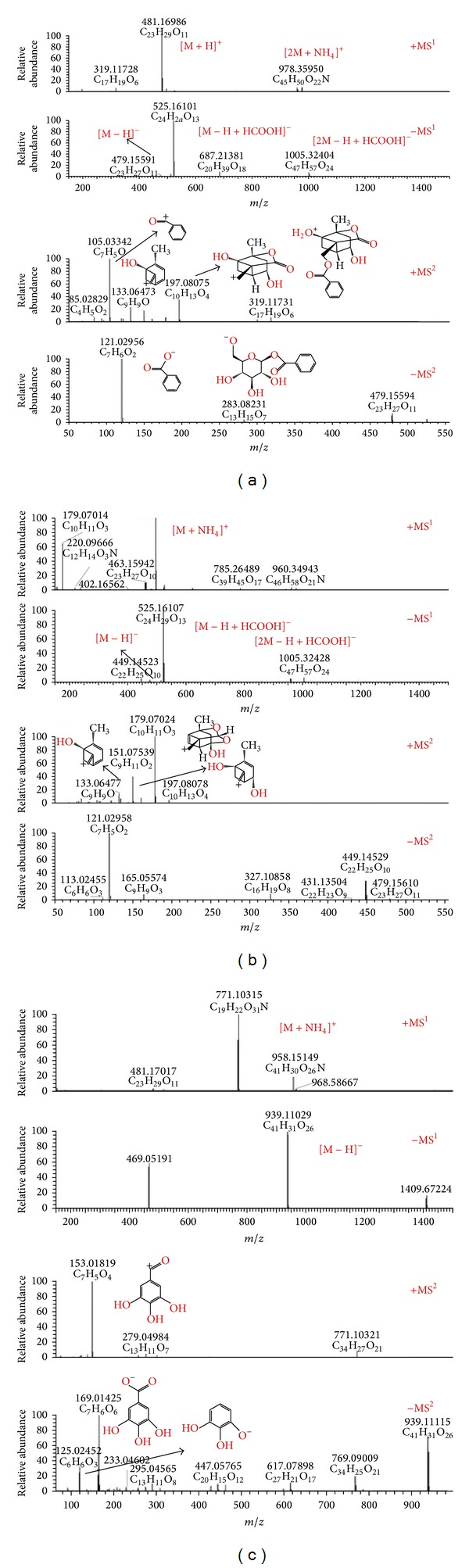
Mass spectra and proposed fragmentations of albiflorin (a), paeoniflorin (b), and 1, 2, 3, 4, 6-penta-O-galloyl-beta-D-glucopyranose (c).

From ESI-MS information, it was found that the sensitivities for all kinds of components in Paeoniae Radix Alba were high in both positive and negative ion modes. In present study, we chose peaks 1, 2, and 3 to explain the identification process using Q Exactive high-performance benchtop quadrupole-Orbitrap LC-MS/MS. Peaks 1, 2, and 3 were eluted at retention times of 4.08, 4.79, and 8.47 min, respectively. Peak 1 showed the [M+H]^+^ *m*/*z* 481.16986, [2 M+NH_4_]^+^ *m*/*z* 978.35950, [M–H]^−^ *m*/*z* 479.15591, [M–H+HCOOH]^−^ *m*/*z* 525.16101, and [2 M−H+HCOOH]^−^ *m*/*z* 1005.32404 and the corresponding elemental compositions were C_23_H_29_O_11_, C_46_H_60_O_22_N, C_23_H_27_O_11_, C_24_H_29_O_13_, and C_47_H_57_O_24_, respectively. On the basis of above data we deduced that the elemental composition of peak 1 was C_23_H_28_O_11_. The molecular ion of peak 1 could lead to seven main MS^2^ ions at* m*/*z* 319.11731, 197.08075, 133.06473, and 105.03342 in positive ion mode, and* m*/*z* 479.15594, 283.08231, and 121.02956 in negative ion mode. On the basis of the elemental compositions of fragment ions, peak 1 was assigned as albiflorin. Peaks 2 and 3 were therefore identified as paeoniflorin, and 1, 2, 3, 4, 6-penta-O-galloyl-beta-D-glucopyranose with above mentioned method. The mass spectra and proposed fragmentations of albiflorin, paeoniflorin, and 1, 2, 3, 4, 6-penta-O-galloyl-beta-D-glucopyranose were shown in Figure 2.

### 3.2. Identification of the Main Components in Crude and Processed Atractylodis Macrocephalae Rhizoma

**Figure 3:**
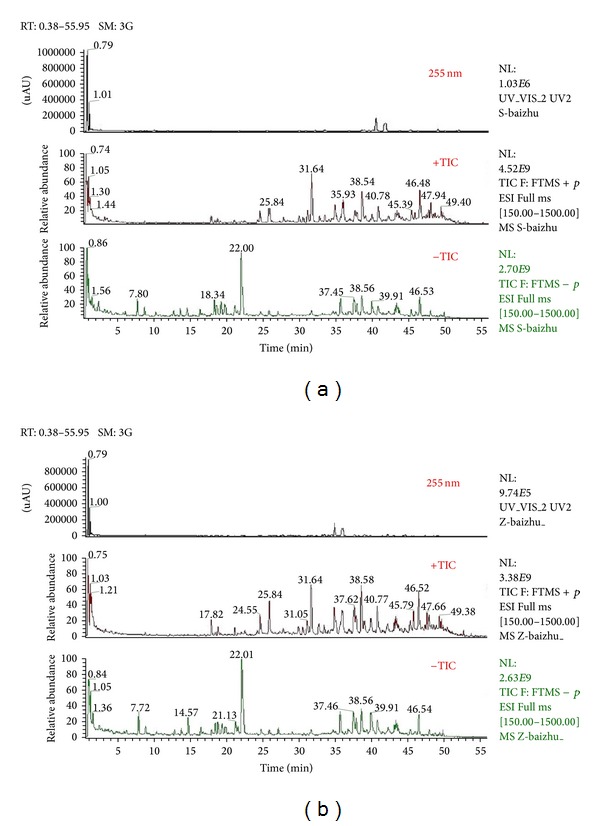
Total ion chromatograms of crude (a) and processed (b) Atractylodis Macrocephalae Rhizoma obtained from both positive and negative ion modes.

Figure 3 showed the total ion chromatograms of crude and processed Atractylodis Macrocephalae Rhizoma samples obtained from both positive and negative ion modes. 32 and 26 compounds were identified in crude and processed Atractylodis Macrocephalae Rhizoma samples, respectively. Compounds 2, 4, 13, 14, 17, and 29 were not detected in processed Atractylodis Macrocephalae Rhizoma sample. Moreover, the amounts of compounds 3, 7, 9, 10, 21, 23, and 27 were substantially decreased, and the amounts of compounds 8, 18, and 22 were increased in processed sample compared with crude one. The results were shown in Table 2.

**Figure 4:**
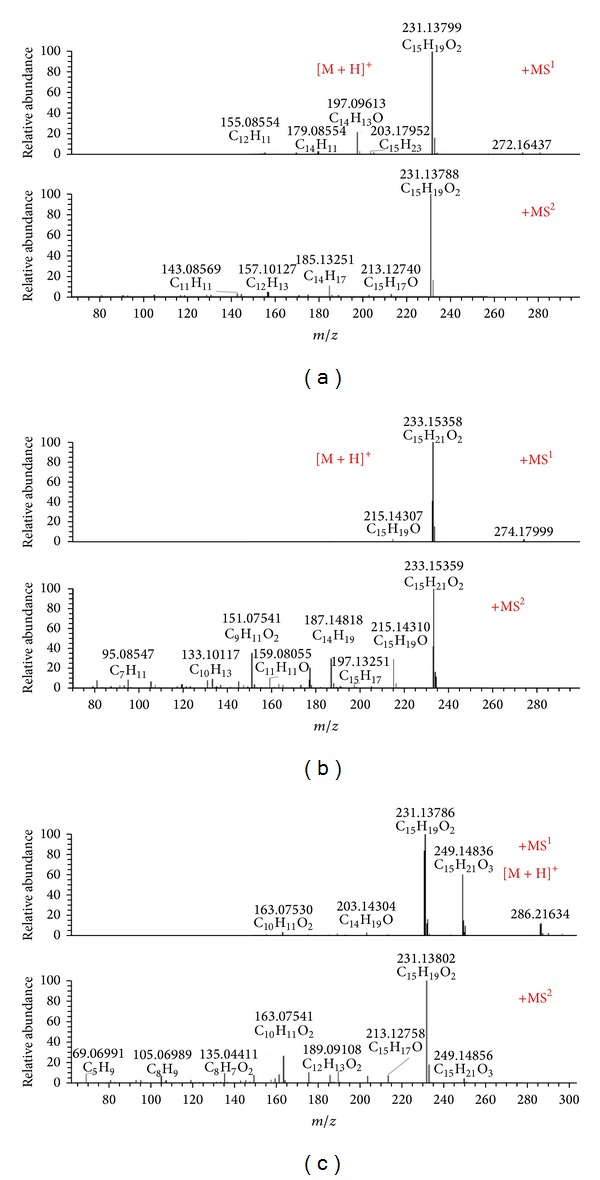
Mass spectra of atractylenolide I (a), atractylenolide II (b), and atractylenolide III (c).

Atractylenolide I, atractylenolide II, and atractylenolide III are the main active compounds that belong to the sesquiterpenes in Atractylodis Macrocephalae Rhizoma. The mass spectra of atractylenolide I showed a [M+H]^+^ ion at* m*/*z* 231.13799, which could lead to four MS^2^ ions at* m*/*z* 213.12740, 185.13251, 157.10127, and 143.08569. The molecular ion of atractylenolide II ([M+H]^+^ *m*/*z* 233.15358) could lead to six MS^2^ ions at* m*/*z* 215.14310, 187.14818, 159.08055, 151.07541, 133.10117, and 95.08547. Meanwhile, the MS^2^ spectrum of* m*/*z* 249.14836 from atractylenolide III contained six major fragment ions at* m*/*z* 231.13802, 213.12758, 189.09108, 163.07541, 135.04411, and 105.06989. The mass spectra of the above three compounds were shown in Figure 4.

### 3.3. Analysis of Chemical Changes of Paeoniae Radix Alba after Compatibility with Atractylodis Macrocephalae Rhizoma

**Figure 5:**
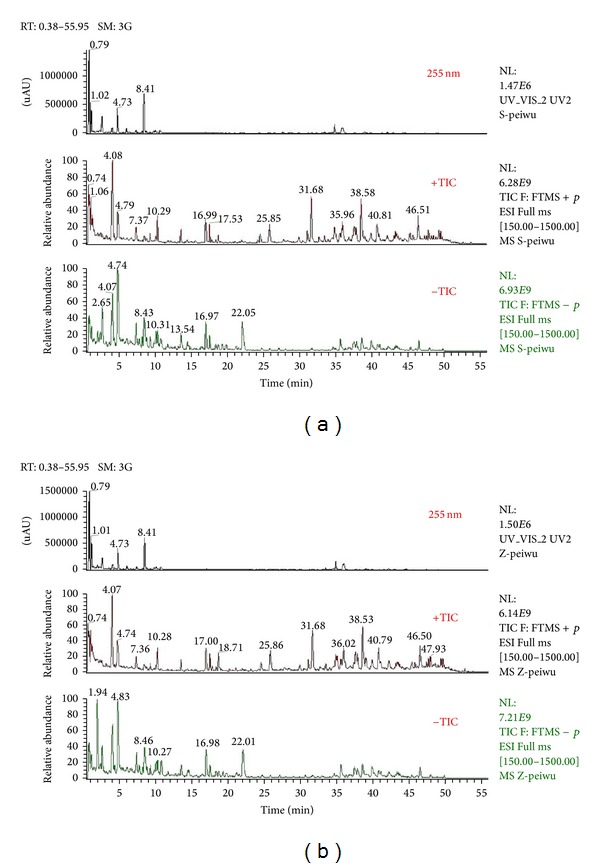
Total ion chromatograms of crude (a) and processed (b) Paeoniae Radix Alba-Atractylodis Macrocephalae Rhizoma herbal pair obtained from both positive and negative ion modes.

In the present study, the Q Exactive high-performance benchtop quadrupole-Orbitrap LC-MS/MS based on chemical profiling approach was used to evaluate chemical constitution between co-decoction and single decoction of Paeoniae Radix Alba and Atractylodis Macrocephalae Rhizoma. For crude Paeoniae Radix Alba, the relative contents of most compounds were dramatically decreased except those of compounds 80, 90, 98, 113, 119, and 122 were significantly increased and 19 compounds were not detected after its compatibility with crude Atractylodis Macrocephalae Rhizoma. For processed Paeoniae Radix Alba, the relative contents of compounds 12, 36, 84, and 86 were remarkably increased except 12 compounds including pedunculagin, oxypaeoniflorin, 6-O-glucopyranosyl-lactinolide, 1, 2, 3, 6-tetra-O-galloylglucose isomer A, 1, 2, 3, 6-tetra-O-galloylglucose isomer B, tetragalloyl glucose C, galloylpaeoniflorin isomer II, hexagalloyl glucose, 3, 6-di-O-galloyl paeoniorin isomer, oxybenzoyl-oxypaeoniflorin, benzoyloxypaeoniflorin, and albiflorin R1 isomer III were newly generated and 13 compounds were not found after its compatibility with processed Atractylodis Macrocephalae Rhizoma. The results were presented in Figure 5 and Table 1.

### 3.4. Analysis of the Chemical Changes of Atractylodis Macrocephalae Rhizoma after Compatibility with Paeoniae Radix Alba

For crude Atractylodis Macrocephalae Rhizoma, the relative contents of compounds 17, 18, and 25 were increased clearly except those of compounds 6, 23, and 30 decreased considerably and six compounds including protocatechuic acid isomer I, protocatechuic acid isomer II, atracetylentriol, 12-methylbutyryl-14-acetyl-2E, 8EZ, 10E-atractylentriol, 12-methylbutyryl-14-acetyl-2E, 8EZ, 10E-atractylentriol isomer, and linoleic acid isomer were lost after its compatibility with crude Paeoniae Radix Alba. For processed Atractylodis Macrocephalae Rhizoma, compounds 9, 20, 26, 27, and 30 were not found except the relative contents of compounds 5, 6, and 8 were decreased while those of compounds 15, 19, 21, and 31 were increased after its compatibility with processed Paeoniae Radix Alba. Furthermore, compound 4 (protocatechuic acid isomer II) was not found in processed Atractylodis Macrocephalae Rhizoma but could be detected in processed Paeoniae Radix Alba-Atractylodis Macrocephalae Rhizoma herbal pair by using Exact Finder and MassFrontier softwares. The above results illustrated that Paeoniae Radix Alba significantly changed the components of Atractylodis Macrocephalae Rhizoma in solution when they decocted together. The corresponding results were presented in Figure 5 and Table 2.

## 4. Conclusions

Q Exactive high-performance benchtop quadrupole-Orbitrap LC-MS/MS is a powerful tool for discriminating the chemical changes between single herbal and co-decocting medicines. In our present study, the Q Exactive high-performance benchtop quadrupole-Orbitrap LC-MS/MS based on chemical profiling approach to investigate and evaluate chemical changes from crude and processed Paeoniae Radix Alba, crude and processed Atractylodis Macrocephalae Rhizoma, and their crude and processed herbal pair extracts was proposed. The results showed that processing and compatibility of TCM could significantly change the chemical composition of Paeoniae Radix Alba and Atractylodis Macrocephalae Rhizoma. The developed method is considered to provide a scientific foundation for deeply elucidating the processing and compatibility mechanism of Paeoniae Radix Alba and Atractylodis Macrocephalae Rhizoma.

## Figures and Tables

**Table 1:** Major chemical constituents identified in crude and processed Paeoniae Radix Alba and in crude and processed Paeoniae Radix Alba-Atractylodis Macrocephalae Rhizoma herbal pair.

No.	*t*_*R*_ (min)	Compound name	Formula	Paeoniae Radix Alba		Paeoniae Radix Alba-Atractylodis Macrocephalae Rhizoma herbal pair	
				(Measured area)		(Measured area)	
				Crude	Processed	Crude	Processed
1	0.84	6-O-galloylsucrose	C_19_H_26_O_15_	1.8570*E* + 08	1.9012*E* + 08	4.2870*E* + 07	4.4158*E* + 07
2	0.84	Glucogallin	C_13_H_16_O_10_	2.9739*E* + 08	2.5698*E* + 08	1.0931*E* + 08	—
3	1.05	Desbenzoylpaeoniflorin	C_16_H_24_O_10_	1.6682*E* + 08	1.6263*E* + 08	9.8500*E* + 07	—
4	1.06	1′-O-galloylsucrose	C_19_H_26_O_15_	3.2574*E* + 08	2.9123*E* + 08	—	—
5	1.07	1-O-glucopyranosyl paeonisuffrone	C_16_H_24_O_9_	2.8667*E* + 08	2.3654*E* + 08	1.1532*E* + 08	—
6	1.13	Gallic acid	C_7_H_6_O_5_	4.1152*E* + 09	4.0736*E* + 09	2.7186*E* + 09	3.1711*E* + 09
7	1.18	Oxypaeoniflorin sulfonate	C_23_H_28_O_14_S	4.9527*E* + 07	3.5407*E* + 07	6.1568*E* + 06	8.5010*E* + 07
8	1.22	Ethyl gallate	C_9_H_10_O_5_	5.1351*E* + 07	6.7200*E* + 07	1.5592*E* + 07	4.8337*E* + 07
9	1.22	6-O-galloyl desbenzoylpaeoniflorin	C_23_H_28_O_14_	9.9020*E* + 07	9.5040*E* + 07	5.3798*E* + 07	—
10	1.26	6-O-glucopyranosyl-lactinolide	C_16_H_26_O_9_	1.0875*E* + 08	1.1180*E* + 08	—	3.7130*E* + 07
11	1.30	Paeoniflorin sulfonate I	C_23_H_28_O_13_S	5.3777*E* + 07	3.6391*E* + 07	7.3077*E* + 06	7.7185*E* + 07
12	1.30	Mudanpioside E sulfonate	C_24_H_30_O_15_S	5.3777*E* + 07	3.6391*E* + 07	7.3077*E* + 06	7.7185*E* + 07
13	1.43	6-O-glucopyranosyl-lactinolide	C_16_H_26_O_9_	7.4342*E* + 08	6.5904*E* + 08	4.0712*E* + 08	3.1407*E* + 08
14	1.64	Mudanpioside F	C_16_H_24_O_8_	6.4178*E* + 08	6.0980*E* + 08	4.0130*E* + 08	6.6680*E* + 07
15	1.76	Isomaltopaeoniflorin sulfonate	C_29_H_38_O_18_S	1.8858*E* + 09	1.1382*E* + 09	2.6277*E* + 08	5.8622*E* + 07
16	1.81	Pedunculagin	C_34_H_24_O_22_	4.8098*E* + 07	—	5.6076*E* + 07	1.2660*E* + 09
17	1.97	Paeoniflorin sulfonate I	C_23_H_28_O_13_S	3.1881*E* + 10	2.3387*E* + 10	6.6202*E* + 09	5.5241*E* + 10
18	2.25	Oxypaeoniflorin	C_23_H_28_O_12_	2.3173*E* + 09	2.4115*E* + 09	1.6734*E* + 09	1.4513*E* + 09
19	2.36	Gallotannin	C_27_H_24_O_18_	2.2850*E* + 08	2.2458*E* + 08	1.6284*E* + 08	1.6703*E* + 08
20	2.37	1-O-benzoylsucrose	C_19_H_26_O_12_	1.3761*E* + 08	1.3161*E* + 08	1.1673*E* + 08	8.2986*E* + 07
21	2.41	d-catechin	C_15_H_14_O_6_	3.7822*E* + 09	4.2278*E* + 09	2.6339*E* + 09	2.5982*E* + 09
22	2.63	Methyl gallate	C_8_H_8_O_5_	2.3823*E* + 10	2.4116*E* + 10	2.3388*E* + 10	—
23	2.63	Salicylic acid	C_7_H_6_O_3_	2.3823*E* + 10	2.4116*E* + 10	2.3388*E* + 10	1.7399*E* + 10
24	2.72	Albiflorin R1	C_23_H_28_O_11_	5.2469*E* + 08	5.6725*E* + 08	5.6329*E* + 08	4.5647*E* + 08
25	3.00	Kaempferol-3,7-di-O-glucoside	C_27_H_30_O_16_	3.8065*E* + 07	2.1896*E* + 07	3.5719*E* + 07	1.5513*E* + 07
26	3.00	Paeonoside	C_27_H_30_O_16_	3.8065*E* + 07	2.1896*E* + 07	3.5719*E* + 07	1.5513*E* + 07
27	3.46	Galloylpaeoniflorin	C_30_H_32_O_15_	1.3912*E* + 08	1.5200*E* + 08	1.1501*E* + 08	1.0863*E* + 08
28	3.47	Paeonolide	C_20_H_28_O_12_	1.0622*E* + 07	1.1936*E* + 07	9.1812*E* + 06	—
29	3.58	6-O-glucopyranosyl-lactinolide	C_16_H_26_O_9_	2.5249*E* + 08	2.3834*E* + 08	2.2675*E* + 08	2.1241*E* + 08
30	3.68	Oxypaeoniflorin	C_23_H_28_O_12_	1.5407*E* + 08	—	1.4345*E* + 08	1.3307*E* + 08
31	3.76	6-O-glucopyranosyl-lactinolide	C_16_H_26_O_9_	3.2664*E* + 08	—	3.0588*E* + 08	3.1627*E* + 08
32	3.88	Paeonilactone B	C_10_H_12_O_4_	9.0325*E* + 07	9.3539*E* + 07	5.1257*E* + 07	8.9597*E* + 07
33	3.93	Isomaltopaeoniflorin	C_29_H_38_O_16_	1.1545*E* + 10	1.1941*E* + 10	1.2282*E* + 10	9.4600*E* + 09
34	4.07	Albiflorin	C_23_H_28_O_11_	2.9587*E* + 10	2.9296*E* + 10	2.8430*E* + 10	2.8684*E* + 10
35	4.32	Glucopyranosylalbiorin	C_29_H_38_O_16_	2.2813*E* + 09	2.4109*E* + 08	2.0383*E* + 08	1.6844*E* + 08
36	4.34	Galloylpaeoniflorin sulfonate	C_30_H_32_O_17_S	7.6943*E* + 08	5.6793*E* + 08	1.5501*E* + 08	1.4886*E* + 09
37	4.34	Galloylpaeoniflorin isomer	C_30_H_32_O_15_	6.7592*E* + 08	7.4322*E* + 08	6.0470*E* + 08	5.4051*E* + 08
38	4.38	1,2,3,6-tetra-O-galloylglucose	C_34_H_28_O_22_	4.6602*E* + 08	3.4977*E* + 08	4.0697*E* + 08	3.6901*E* + 08
39	4.38	Tetragalloyl glucose A	C_34_H_28_O_22_	4.6602*E* + 08	3.4977*E* + 08	4.0697*E* + 08	3.6901*E* + 08
40	4.56	Mudanpioside F	C_16_H_24_O_8_	8.4156*E* + 07	8.2734*E* + 07	7.2666*E* + 07	7.9227*E* + 07
41	4.60	Oxypaeoniflorin isomer	C_23_H_28_O_12_	9.6610*E* + 08	9.8706*E* + 08	9.2464*E* + 08	8.7359*E* + 08
42	4.65	Gallotannin	C_27_H_24_O_18_	6.7737*E* + 07	—	—	—
43	4.77	Paeoniflorin	C_23_H_28_O_11_	5.9556*E* + 10	6.1356*E* + 10	5.9929*E* + 10	5.8832*E* + 10
44	4.89	Paeoniflorin sulfonate II	C_23_H_28_O_13_S	1.1095*E* + 08	1.4567*E* + 08	5.5052*E* + 07	2.7813*E* + 08
45	4.98	Isogalloylpaeoniflorin sulfonate	C_30_H_32_O_17_S	3.6742*E* + 07	—	—	—
46	5.05	Ethyl gallate	C_9_H_10_O_5_	6.0669*E* + 07	5.4850*E* + 07	1.6681*E* + 07	2.6790*E* + 07
47	5.05	Methyl salicylate	C_8_H_8_O_3_	6.0669*E* + 07	5.4850*E* + 07	1.6681*E* + 07	2.6790*E* + 07
48	5.15	Benzoic acid	C_7_H_6_O_2_	4.0163*E* + 07	4.5493*E* + 07	2.9695*E* + 07	2.9727*E* + 07
49	5.25	Paeonol	C_9_H_10_O_3_	6.8567*E* + 07	7.4129*E* + 07	9.9992*E* + 07	6.5619*E* + 07
50	5.25	4-hydroxy-3-methoxy acetophenone	C_9_H_10_O_3_	6.8567*E* + 07	7.4129*E* + 07	9.9992*E* + 07	6.5619*E* + 07
51	5.31	ortho-oxypaeoniflorin	C_23_H_28_O_12_	1.9080*E* + 09	1.9263*E* + 09	1.8723*E* + 09	1.6842*E* + 09
52	5.63	Ethyl gallate	C_9_H_10_O_5_	1.4627*E* + 08	1.2812*E* + 08	1.0365*E* + 08	8.8155*E* + 07
53	5.63	Methyl salicylate	C_8_H_8_O_3_	1.4627*E* + 08	1.2812*E* + 08	1.0365*E* + 08	8.8155*E* + 07
54	5.66	Kaempferol-3-O-glucoside	C_21_H_20_O_11_	1.6012*E* + 07	1.7385*E* + 07	—	—
55	5.66	Astragalin	C_21_H_20_O_11_	1.6012*E* + 07	1.7385*E* + 07	—	—
56	6.01	Eugeniin	C_41_H_30_O_26_	2.7483*E* + 08	3.0279*E* + 08	2.8080*E* + 08	3.0479*E* + 08
57	6.01	Dihydroxymethyl benzoyl tetragalloyl glucose	C_41_H_30_O_26_	2.7483*E* + 08	3.0279*E* + 08	2.8080*E* + 08	3.0479*E* + 08
58	6.03	1,2,3,6-tetra-O-galloylglucose isomer A	C_34_H_28_O_22_	1.3555*E* + 09	—	1.1980*E* + 09	1.1039*E* + 09
59	6.03	Tetragalloyl glucose B	C_34_H_28_O_22_	1.3555*E* + 09	—	—	—
60	6.08	Astragalin	C_21_H_20_O_11_	1.5009*E* + 07	1.8552*E* + 07	1.5922*E* + 07	1.4002*E* + 07
61	6.09	Isomaltopaeoniflorin isomer	C_29_H_38_O_16_	7.5172*E* + 07	—	—	—
62	6.47	1,2,3,6-tetra-O-galloylglucose isomer B	C_34_H_28_O_22_	1.5882*E* + 09	—	—	1.2570*E* + 09
63	6.47	Tetragalloyl glucose C	C_34_H_28_O_22_	1.5882*E* + 09	—	—	1.2570*E* + 09
64	6.85	3,6-di-O-galloyl paeoniorin	C_37_H_36_O_19_	7.6512*E* + 07	—	—	—
65	6.96	1,2,3,6-tetra-O-galloylglucose	C_34_H_28_O_22_	4.4729*E* + 08	4.5825*E* + 08	4.0642*E* + 08	4.2393*E* + 08
66	6.96	Tetragalloyl glucose D	C_34_H_28_O_22_	4.4729*E* + 08	4.5825*E* + 08	4.0642*E* + 08	4.2393*E* + 08
67	7.35	Galloylpaeoniflorin isomer I	C_30_H_32_O_15_	1.2156*E* + 10	1.2451*E* + 10	1.1484*E* + 10	1.0962*E* + 10
68	7.60	1-O-glucopyranosyl-8-O-benzoyl paeonisuffrone	C_23_H_28_O_10_	4.3983*E* + 07	4.5347*E* + 07	4.4927*E* + 07	3.9869*E* + 07
69	7.71	Glucopyranosylalbiorin isomer I	C_29_H_38_O_16_	7.2982*E* + 07	7.9341*E* + 07	—	1.8872*E* + 07
70	8.18	1-O-glucopyranosyl-8-O-benzoyl paeonisuffrone	C_23_H_28_O_10_	7.4648*E* + 07	8.3204*E* + 07	6.5957*E* + 07	5.9832*E* + 07
71	8.31	Ortho-oxypaeoniflorin	C_23_H_28_O_12_	2.4469*E* + 07	2.4504*E* + 07	2.3932*E* + 07	2.2796*E* + 07
72	8.45	1,2,3,4,6-Penta-O-galloyl–D-glucopyranose	C_41_H_32_O_26_	1.1843*E* + 10	1.0905*E* + 10	1.0518*E* + 10	1.0489*E* + 10
73	8.45	Pentagalloyl glucose	C_41_H_32_O_26_	1.1843*E* + 10	1.0905*E* + 10	1.0518*E* + 10	1.0489*E* + 10
74	8.64	Lactiflorin	C_23_H_26_O_10_	1.0818*E* + 08	1.8628*E* + 08	1.3689*E* + 08	—
75	8.80	Galloylalbiroin	C_30_H_32_O_15_	3.2696*E* + 09	—	—	—
76	9.17	Astragalin	C_21_H_20_O_11_	1.0717*E* + 07	1.3960*E* + 07	1.2843*E* + 07	1.0582*E* + 07
77	9.25	Lactinolide	C_10_H_16_O_4_	2.7251*E* + 07	2.6105*E* + 07	2.1735*E* + 07	3.2770*E* + 07
78	9.29	Galloylpaeoniflorin isomer II	C_30_H_32_O_15_	2.8831*E* + 09	—	2.6829*E* + 09	2.2850*E* + 09
79	9.68	Glucopyranosylalbiorin isomer II	C_29_H_38_O_16_	2.4321*E* + 07	2.6804*E* + 07	2.2576*E* + 07	2.4950*E* + 07
80	9.84	Hexagalloyl glucose	C_48_H_36_O_30_	4.9153*E* + 07	—	6.8676*E* + 08	5.7793*E* + 08
81	9.95	Oxybenzoyl-oxypaeoniflorin	C_30_H_32_O_14_	1.4385*E* + 07	1.6654*E* + 07	1.1051*E* + 07	1.1345*E* + 07
82	10.07	1-O-glucopyranosyl-8-O-benzoylpaeonisuffrone	C_23_H_28_O_10_	3.6916*E* + 09	3.5634*E* + 09	3.1333*E* + 09	3.2106*E* + 09
83	10.29	Albiflorin R1 isomer I	C_23_H_28_O_11_	6.3346*E* + 09	6.6205*E* + 09	5.9528*E* + 09	5.8736*E* + 09
84	10.74	Hexagalloyl glucose	C_48_H_36_O_30_	4.9225*E* + 08	2.5582*E* + 08	1.9395*E* + 09	1.5439*E* + 09
85	10.76	Lactiflorin	C_23_H_26_O_10_	1.2785*E* + 09	3.5174*E* + 09	9.9713*E* + 08	3.4524*E* + 09
86	10.84	Benzoylpaeoniflorin Sulfonate	C_30_H_32_O_14_S	9.0616*E* + 08	6.4075*E* + 08	1.5946*E* + 08	2.1931*E* + 09
87	10.88	3,6-di-O-galloyl paeoniorin	C_37_H_36_O_19_	1.6123*E* + 08	—	—	—
88	10.95	Ortho-oxypaeoniflorin isomer	C_23_H_28_O_12_	5.5563*E* + 07	5.8774*E* + 07	5.7147*E* + 07	5.6640*E* + 07
89	11.52	3,6-di-O-galloyl paeoniorin	C_37_H_36_O_19_	3.6509*E* + 08	3.9290*E* + 08	5.2162*E* + 08	5.3781*E* + 08
90	11.72	3,6-di-O-galloyl paeoniorin isomer	C_37_H_36_O_19_	9.7356*E* + 08	—	1.2523*E* + 09	9.5929*E* + 08
91	11.75	Galloylalbiroin isomer I	C_30_H_32_O_15_	2.3457*E* + 08	—	—	—
92	11.84	Oxypaeoniflorin sulfonate isomer	C_23_H_28_O_14_S	2.1063*E* + 07	1.9747*E* + 07	1.3875*E* + 07	1.0840*E* + 07
93	12.15	1-O-glucopyranosyl-8-O-benzoylpaeonisuffrone	C_23_H_28_O_10_	7.2104*E* + 07	7.1468*E* + 07	6.7309*E* + 07	6.5917*E* + 07
94	12.15	Oxybenzoyl-oxypaeoniflorin	C_30_H_32_O_14_	1.9982*E* + 08	—	—	1.6891*E* + 08
95	12.18	Benzoyloxypaeoniflorin	C_30_H_32_O_13_	2.0822*E* + 08	—	2.0163*E* + 08	1.9074*E* + 08
96	13.42	Benzoyloxypaeoniflorin isomer	C_30_H_32_O_13_	8.6458*E* + 07	6.2282*E* + 07	7.6048*E* + 07	7.2791*E* + 07
97	13.44	Oxybenzoyl-oxypaeoniflorin isomer I	C_30_H_32_O_14_	1.4728*E* + 07	1.7389*E* + 07	1.5360*E* + 07	1.6008*E* + 07
98	13.85	Galloylalbiroin isomer II	C_30_H_32_O_15_	9.6403*E* + 07	1.2196*E* + 08	1.0506*E* + 08	1.0272*E* + 08
99	14.05	Oxybenzoyl-oxypaeoniflorin isomer II	C_30_H_32_O_14_	2.5323*E* + 07	2.9603*E* + 07	2.3556*E* + 07	2.8526*E* + 07
100	14.13	Benzoyloxypaeoniflorin	C_30_H_32_O_13_	3.8096*E* + 07	3.8557*E* + 07	3.7499*E* + 07	3.5800*E* + 07
101	15.07	Benzoyloxypaeoniflorin isomer I	C_30_H_32_O_13_	1.9827*E* + 07	2.3616*E* + 07	—	—
102	15.38	Benzoyloxypaeoniflorin isomer II	C_30_H_32_O_13_	1.1841*E* + 07	1.3730*E* + 07	—	—
103	16.01	Oxybenzoyl-paeoniflorin	C_30_H_32_O_12_	1.8152*E* + 07	—	1.8435*E* + 07	—
104	16.95	Isobenzoylpaeoniflorin	C_30_H_32_O_12_	1.2225*E* + 10	1.3228*E* + 10	1.2158*E* + 10	1.2391*E* + 10
105	16.95	Oxybenzoyl-paeoniflorin isomer I	C_30_H_32_O_12_	1.2225*E* + 10	1.3228*E* + 10	1.2158*E* + 10	1.2391*E* + 10
106	17.23	Benzoylpaeoniflorin Sulfonate	C_30_H_32_O_14_S	1.5680*E* + 07	1.2235*E* + 07	5.6573*E* + 06	3.5831*E* + 07
107	17.48	Isobenzoylpaeoniflorin isomer I	C_30_H_32_O_12_	5.4138*E* + 09	5.4432*E* + 09	5.2522*E* + 09	5.3238*E* + 09
108	17.48	Oxybenzoyl-paeoniflorin isomer II	C_30_H_32_O_12_	5.4138*E* + 09	5.4432*E* + 09	5.2522*E* + 09	5.3238*E* + 09
109	17.86	Benzoyloxypaeoniflorin	C_30_H_32_O_13_	3.4347*E* + 07	3.4852*E* + 07	3.5980*E* + 07	3.8814*E* + 07
110	18.55	Benzoyloxypaeoniflorin isomer	C_30_H_32_O_13_	1.5397*E* + 07	1.7656*E* + 07	1.7246*E* + 07	1.8012*E* + 07
111	18.69	Albiflorin R1 isomer II	C_23_H_28_O_11_	2.0046*E* + 07	1.9851*E* + 07	2.3462*E* + 07	—
112	19.30	Albiflorin R1 isomer III	C_23_H_28_O_11_	2.9827*E* + 06	—	—	5.6105*E* + 06
113	21.79	Palbinone	C_22_H_30_O_4_	8.9687*E* + 07	1.3174*E* + 08	1.2834*E* + 08	5.7610*E* + 07
114	21.93	Isobenzoylpaeoniflorin isomer II	C_30_H_32_O_12_	4.5356*E* + 08	4.2874*E* + 07	3.4016*E* + 08	2.7347*E* + 08
115	21.93	Oxybenzoyl-paeoniflorin isomer III	C_30_H_32_O_12_	4.5356*E* + 08	4.2874*E* + 07	3.4016*E* + 08	2.7347*E* + 08
116	22.15	Paeonilactinone	C_10_H_16_O_2_	7.0423*E* + 06	3.7108*E* + 06	8.0036*E* + 06	6.6886*E* + 06
117	36.46	Hederagenin	C_30_H_48_O_4_	7.6725*E* + 07	8.1456*E* + 07	9.7498*E* + 07	4.7332*E* + 07
118	37.31	23-hydroxybetulinic acid	C_30_H_48_O_4_	3.9836*E* + 07	4.0995*E* + 07	3.9906*E* + 07	2.2611*E* + 07
119	38.14	Astrantiagenin D	C_30_H_46_O_4_	7.8714*E* + 06	7.9560*E* + 06	1.1904*E* + 07	3.8958*E* + 06
120	43.00	Astrantiagenin D isomer	C_30_H_46_O_4_	4.0450*E* + 06	—	3.1585*E* + 06	—
121	45.65	Oleanolic acid	C_30_H_48_O_3_	1.1266*E* + 08	9.4258*E* + 07	7.6434*E* + 07	4.3295*E* + 07
122	46.10	Betulinic acid	C_30_H_48_O_3_	6.2494*E* + 06	2.3289*E* + 07	4.0543*E* + 07	2.3912*E* + 07
123	52.48	Daucosterol	C_35_H_60_O_6_	1.4060*E* + 07	1.9624*E* + 07	8.5440*E* + 06	6.3156*E* + 06

**Table 2:** Major chemical constituents identified in crude and processed Atractylodis Macrocephalae Rhizoma and in crude and processed Paeoniae Radix Alba-Atractylodis Macrocephalae Rhizoma herbal pair.

No.	*t*_*R*_ (min)	Compound name	Formula	Atractylodis Macrocephalae Rhizoma		Paeoniae Radix Alba-Atractylodis Macrocephalae Rhizoma herbal pair	
				(Measured area)		(Measured area)	
				Crude	Processed	Crude	Processed
1	1.72	Protocatechuic acid	C_7_H_6_O_4_	2.0389*E* + 07	1.4454*E* + 07	2.0881*E* + 07	2.4383*E* + 07
2	2.67	Protocatechuic acid isomer I	C_7_H_6_O_4_	9.6661*E* + 07	—	—	—
3	3.24	Caffeic acid	C_9_H_8_O_4_	3.6818*E* + 08	1.7393*E* + 08	2.8796*E* + 08	1.2882*E* + 08
4	3.73	Protocatechuic acid isomer II	C_7_H_6_O_4_	2.0846*E* + 07	—	—	1.2022*E* + 07
5	4.21	Dictamnoside A isomer I	C_21_H_36_O_9_	1.8843*E* + 07	2.4981*E* + 07	1.0636*E* + 07	1.3140*E* + 07
6	4.70	Dictamnoside A isomer II	C_21_H_36_O_9_	2.8770*E* + 07	3.4768*E* + 07	1.0395*E* + 07	1.4208*E* + 07
7	5.63	Scopoletin	C_10_H_8_O_4_	6.1458*E* + 07	4.1494*E* + 07	6.1562*E* + 07	5.3342*E* + 07
8	5.82	Dictamnoside A	C_21_H_36_O_9_	9.6195*E* + 07	1.1991*E* + 08	7.5446*E* + 07	9.4190*E* + 07
9	8.77	Atracetylentriol	C_14_H_16_O_3_	1.2538*E* + 07	5.4052*E* + 06	—	—
10	9.33	Ferulic acid	C_10_H_10_O_4_	1.3958*E* + 07	9.1214*E* + 06	1.1912*E* + 07	9.6849*E* + 06
11	25.81	Atractylenolide I isomer	C_15_H_18_O_2_	4.5224*E* + 09	4.2401*E* + 09	5.9401*E* + 09	6.5277*E* + 09
12	25.83	Atractylenolide III	C_15_H_20_O_3_	2.5549*E* + 09	1.8023*E* + 09	2.8280*E* + 09	3.1632*E* + 09
13	26.17	12-methylbutyryl-14-acetyl-2E,8EZ,10E-atractylentriol	C_21_H_26_O_5_	2.4755*E* + 07	—	—	—
14	26.95	12-methylbutyryl-14-acetyl-2E,8EZ,10E-atractylentriol isomer	C_21_H_26_O_5_	7.5991*E* + 07	—	—	—
15	31.10	Atractylenolide II isomer	C_15_H_20_O_2_	6.7883*E* + 09	4.5794*E* + 09	7.6246*E* + 09	7.8814*E* + 09
16	31.66	Atractylenolide II	C_15_H_20_O_2_	2.8279*E* + 10	1.9902*E* + 10	3.0285*E* + 10	3.1294*E* + 10
17	33.44	Atractylodin	C_13_H_10_O	6.4157*E* + 06	—	7.0452*E* + 07	—
18	35.07	Atractylenolide I isomer	C_15_H_18_O_2_	8.2226*E* + 08	1.4781*E* + 09	1.0831*E* + 09	3.2083*E* + 09
19	35.94	Atractylenolide I	C_15_H_18_O_2_	8.8877*E* + 09	7.2520*E* + 09	8.3857*E* + 09	1.2742*E* + 10
20	39.03	12-methylbutyryl-14-acetyl-2E,8EZ,10E-atractylentriol isomer I	C_21_H_26_O_5_	3.0978*E* + 07	3.7863*E* + 07	2.9171*E* + 07	—
21	39.81	Dibutyl phthalate	C_16_H_22_O_4_	1.1372*E* + 08	9.8325*E* + 07	1.2659*E* + 08	1.4865*E* + 08
22	40.00	12-methylbutyryl-14-acetyl 2E,8EZ,10E-atractylentriol isomer II	C_21_H_26_O_5_	3.8810*E* + 07	7.7498*E* + 07	3.3885*E* + 07	7.0522*E* + 07
23	40.26	Dibutyl phthalate isomer	C_16_H_22_O_4_	1.0631*E* + 08	5.4902*E* + 07	6.1958*E* + 07	4.6227*E* + 07
24	41.50	14-methylbutyryl-2E,8EZ,10Es-atractylentriol	C_19_H_24_O_4_	4.9587*E* + 07	2.8423*E* + 07	5.1146*E* + 07	4.7855*E* + 07
25	46.43	Spinasteryl	C_29_H_48_O	8.6778*E* + 06	7.9096*E* + 06	1.0609*E* + 07	7.7832*E* + 06
26	47.32	Atractylon	C_15_H_20_O	7.4433*E* + 07	5.4063*E* + 07	6.6146*E* + 07	—
27	47.37	Biatractylolide	C_30_H_38_O_4_	1.0949*E* + 09	9.5665*E* + 08	1.2797*E* + 09	—
28	47.96	Linoleic acid	C_18_H_32_O_2_	1.8499*E* + 08	1.5041*E* + 08	1.8777*E* + 08	2.3743*E* + 08
29	48.25	Linoleic acid isomer	C_18_H_32_O_2_	2.1059*E* + 07	—	—	—
30	48.59	Biepiasterolid isomer	C_30_H_38_O_4_	9.0255*E* + 08	7.0863*E* + 08	7.4011*E* + 08	—
31	48.90	Atractylon isomer	C_15_H_20_O	9.5308*E* + 07	8.7683*E* + 07	8.2967*E* + 07	1.0132*E* + 08
32	49.42	Palmitic acid	C_16_H_32_O_2_	2.2356*E* + 07	2.2942*E* + 07	2.5949*E* + 07	2.0153*E* + 07
